# The ECOS-16 questionnaire for the evaluation of health related quality of life in post-menopausal women with osteoporosis

**DOI:** 10.1186/1477-7525-2-41

**Published:** 2004-08-03

**Authors:** Xavier Badia, Adolfo Díez-Pérez, Raquel Lahoz, Luis Lizán, Xavier Nogués, Jordi Iborra

**Affiliations:** 1 Health Outcomes Research (HOR) Europe, Plató 6, 1° 5^a^, 08021 Barcelona, Spain; 2 Departament d'Epidemiologia I Salut Pública de l'Hospital de la Santa Creu i Sant Pau, Barcelona, Spain; 3 Servei de Medicina Interna, Hospital Ntra. Sra. del Mar, Universitat Autònoma de Barcelona, Barcelona, Spain; 4 Unidad Docente de Medicina de Familia, Castellón, Spain; 5 Novartis Farmacéutica, Barcelona, Spain

## Abstract

**Background:**

The aim of this study is to validate the questionnaire ECOS-16 (Assessment of health related quality of life in osteoporosis) for the evaluation of health related quality of life (HRQoL) in post-menopausal women with osteoporosis.

**Methods:**

An observational, prospective and multi-centre study was carried out among post-menopausal women with osteoporosis in primary care centres and hospital outpatient clinics. All patients attended 2 visits: at baseline and at 6 months. In addition, the subgroup of outpatients attended another visit a month after the baseline to assess the test-retest reliability. The psychometric properties of the questionnaire were evaluated in terms of feasibility, validity (content validity and construct validity) and internal consistency in baseline, and in terms of test-retest reliability and responsiveness to change in visit at month and visit at 6 months, respectively. In all visits, ECOS-16, EUROQoL-5D (EQ-5D) and four 7-point items about health status (general health status, back pain, limitation in daily activities and emotional status) were administered, whereas only outpatients were given MINI-OQLQ (Mini Osteoporosis Quality of Life Questionnaire), besides all clinical variables; and sociodemographic variables at baseline.

**Results:**

316 women were consecutively included, 212 from primary care centres and 104 from hospital outpatient clinics. *Feasibility*: 94.3% of patients answered all items of the questionnaire. The mean administration time was 12.3 minutes. *Validity*: factor analysis suggested that the questionnaire was unidimensional. In the multivariate analysis, patients with vertebral fractures, co-morbidity and a lower education level showed to have worse HRQoL. Moderate to high correlations were found between the ECOS-16 score and the other health status questionnaires (0.47–0.82). *Reliability*: internal consistency (Cronbach's α) was 0.92 and test-retest reliability (ICC) was 0.80. *Responsiveness to change*: ECOS-16 scores increased according to change perceived by the patient, as well as the effect size (ranges between 1.35 to 0.43), the greater the perception of change in patients' general health status, the greater the changes in patients' scores. The Minimal Clinically Important Difference (MCID) suggested a change of 0.5 points in the ECOS-16 score, representing the least improvement in general health status due to their osteoporosis: "slightly better".

**Conclusion:**

ECOS-16 has been proven preliminarily to have good psychometric properties, so that it can be potentially a useful tool to evaluate HRQoL of post-menopausal women with osteoporosis in research and routine clinical practice.

## Background

Osteoporosis is characterised by low bone mass and a deterioration in bone tissue micro-architecture, leading to increasing bone weakness and consequently risk of fracture. The most common clinical complications of osteoporosis are hip fracture, vertebral deformity and wrist fracture. According to bone densitometry values, osteoporosis affects approximately 2 million women in Spain [1,2]. The most frequent symptom of osteoporosis is low back pain resulting from vertebral fractures. This pain can have a considerable impact on the ability to carry out usual activities of daily living. Patients are unable to work normally, are limited in their social and leisure activities, and may be severely affected emotionally [3].

To date, clinical trials on osteoporosis have been based on outcomes measured by imaging tests. But these measurements do not adequately reflect the extent to which the patient is affected in their usual daily activities, and are not appropriate to assess patients' disability and symptoms [4]. Nevertheless, recently some specific Health Related Quality of Life (HRQoL) questionnaires such as OPAQ (Osteoporosis Assessment Questionnaire) have been used as the main outcome in clinical studies on osteoporosis [5]. Several generic HRQoL questionnaires, such as the Sickness Impact Profile (SIP), SF-36 or the Nottingham Health Profile (NHP), have been used more frequently to assess the impact of osteoporosis on HRQoL [6]. These questionnaires are applicable to any population and disease, thereby enabling comparison between subjects suffering from different diseases. However, they have serious limitations given the fact that they fail to explore in detail the specific aspects of osteoporosis. For instance, some studies have shown that certain aspects, such as the fear of falling and suffering a bone fracture, the inability to adequately carry out domestic tasks, the ability to dress oneself adequately without help and despair about an uncertain future are all stressful for these patients [7]. These items are not included in generic questionnaires and their omission could lead to an incomplete or biased evaluation of HRQoL of patients with osteoporosis.

Disease specific questionnaires for osteoporosis are available, such as the Osteoporosis Quality of Life Questionnaire (OQLQ) [8] or the Quality of Life Questionnaire of the European Foundation for Osteoporosis (QUALEFFO) [9]. However, their limited applicability due to their length and time for administration have restricted their use to clinical trials and highlighted the need for the development of questionnaires which are easier to administer in routine clinical practice. In order to expand their use in clinical practice, it is necessary to develop valid, short, easy to administer and comprehensible questionnaires. For this reason, a specific short form HRQoL questionnaire for women with osteoporosis was developed [10]. Its items were obtained from the Spanish versions of the OQLQ and QUALEFFO questionnaires, and were then reduced by using the Rasch analysis to obtain a total of 16 items; 12 from the QUALEFFO and 4 from the OQLQ. These questionnaires were selected because they were the only questionnaires already validated in Spain. More information regarding the development process of the ECOS-16 has been published elsewhere [11,12].

The aim of the present study is to evaluate the psychometric properties of the ECOS-16 in post-menopausal women with osteoporosis.

## Methods

316 post-menopausal women with primary osteoporosis attended in Primary Care Centres or in outpatient clinics were included in the study. Diagnosis was confirmed by a Bone Mineral Density (BMD) using Dual Energy X-Ray Absorptiometry (DEXA). Furthermore, outpatients should have at least one prevalent vertebral fracture confirmed by Genant's radiological criteria due to osteoporosis, a requirement which was not essential in patients from Primary Care Centres. 212 patients from 49 Primary Care Centres and 104 patients attended in outpatient clinics from 14 hospitals were consecutively selected and evaluated from March 2000 to August 2001.

All patients attended two visits, a baseline and a follow-up visit 6 months after the inclusion. In order to evaluate test-retest reliability, outpatients were also attended in another follow-up visit one month after the baseline. All patients received the study information and gave their informed consent.

### Study design

An observational, prospective and multi-centre study was carried out for the validation of the ECOS-16 in post-menopausal women with vertebral fractures due to osteoporosis in conditions of clinical practice. At the baseline, feasibility together with the content and construct validity of the ECOS-16 were evaluated. At the visit after 6 months, responsiveness to change with regard to ECOS-16 was evaluated. Outpatients also attended another visit a month after the inclusion so that the test-retest reliability of the ECOS-16 could be evaluated.

In the baseline, data on the patients' sociodemographic characteristics (age, education level) and clinical variables (weight, height, body mass index, age at onset of menopause, BMD, presence and site of vertebral and non-vertebral fractures, concomitant chronic diseases and received treatment) were collected as well as the ECOS-16, the EUROQoL-5D and four 7-point items which refer to general health status, back pain, limitation in daily activities and emotional status. These items were used in a previous study and showed its validity [10]. Outpatients were also administered the Spanish version of the MINI-OQLQ questionnaire [3].

In the two follow-up visits, any modifications to the specific treatment for osteoporosis prescribed in the baseline, the number of concomitant treatments, and patients' withdrawals causes were registered. Moreover, the ECOS-16, EUROQoL-5D and the four 7-point change items were again administered. Outpatients were again administered the MINI-OQLQ questionnaire.

In the present study, the EQ-5D and MINI-OQLQ questionnaires were used in order to assess the validity of the ECOS-16.

### Health related quality of life questionnaires

#### ECOS-16

The ECOS-16 (Please see additional file 1 ([appendix]) was developed with the aim of measuring HRQoL in postmenopausal women with osteoporosis. It is based on the combination of two disease-specific HRQoL questionnaires for women with osteoporosis: the Osteoporosis Quality of Life Questionnaire (OQLQ) [8] and the Quality of Life Questionnaire of the European Foundation for Osteoporosis (QUALEFFO) [9]. The development process consisted in five phases: Phase I-Search for common structures; Phase II-Independent OQLQ and QUALEFFO item reduction using Rasch analysis; Phase III-OQLQ and QUALEFFO item equating; Phase IV-Quantitative reduction of equated items; Phase V-Qualitative reduction [11]. This newly questionnaire consists of 12 items from the QUALEFFO and 4 from the OQLQ (see Annex 1). All items have five possible response options, although the response options differ from one item to another. The 16 items in the new questionnaire are divided qualitatively into four dimensions. The nature of the four dimensions also suggests that they can be further combined to produce two summary scores that would include Physical Function and Pain in one Physical score, and another one that would include Fear of Illness and Psychosocial Function in a Mental score. These two summary scores could, in turn, be combined to provide an overall score for the questionnaire. However, although the 16 items can be classified qualitatively into four dimensions, this is an unidimensional questionnaire, according to the quantitative analysis [11]. The score of each item ranges from 1 to 5. ECOS-16 generates a single summary score obtained from the arithmetic mean of the answered items, so the total score ranges from 1 (best HRQoL) to 5 (worst HRQoL).

The time frame for the questionnaire was one week. All items have the same weight on the overall questionnaire score and the overall score is calculated as the mean score of all the response items.

It is a self-administered questionnaire, apart from some special cases (eyesight difficulties or illiteracy) where it was acceptable for the questionnaire to be administered by health care personnel or experienced interviewers.

#### EUROQoL-5D

The EUROQoL-5D (EQ-5D) is a generic HRQoL questionnaire. The EQ-5D consists of two parts: a descriptive system and a Visual Analogue Scale (VAS) [13]. The descriptive system contains 5 health status dimensions: Mobility, Self-Care, Usual activities, Pain/Discomfort and Anxiety/Depression. These dimensions are always presented in the same order, each one with 3 degrees of severity: no problems, some or moderate problems, and extreme problems, given a value of 1, 2 and 3, respectively. For each dimension, the respondent should mark the degree of severity which best describes their actual health status.

The VAS of the EQ-5D is a vertical scale divided into millimetres along a 20 centimetres long thermometer where the two ends are labelled "worst imaginable health state" and "best imaginable health state" with a score of 0 and 100, respectively. The respondent should mark the point on the thermometer which, in their opinion, best describes their actual overall health status.

#### MINI-OQLQ

The MINI-OQLQ is a specific HRQoL questionnaire for women with vertebral fractures due to osteoporosis. The MINI-OQLQ is based on a selection of the two highest impact items from each of the five domains of the Osteoporosis Quality of Life Questionnaire (OQLQ). The MINI-OQLQ is, therefore, composed of 10 items grouped into the same five HRQoL dimensions (symptoms, physical function, activities of daily living, emotional function and leisure) [3].

Each item has seven response options ranging from 1 (worse HRQoL) to 7 (better HRQoL). The scoring is obtained per dimension, by calculating the mean score of the response items for each dimension, so that the higher the score in each dimension, the better the resulting HRQoL.

### 7-point change items (general health status, back pain, limitation in daily activities and emotional status) due to osteoporosis

Changes in four health status items were assessed through four different items regarding the change in the patient's overall health status: the change in the patient's general health status due to osteoporosis, the change in the patient's back pain, the change in the patient's limitation in daily activities and the change in the patient's emotional status, all of them due to osteoporosis, with reference to the baseline. The items have seven possible response options, ranging from "Much better" to "Much worse" and including the category "More or less the same". These items were designed to be self-administered and validated in previous studies [10,14].

### Statistical analysis

Double data entry was carried out with a subsequent validation to guarantee the quality and consistency of the data. A statistical significance level of p < 0.05 was used in all statistical tests performed. The statistical program SPSS^® ^for Windows version 10.0 (SPSS, Inc., Chicago, Illinois) was used to carry out the entire data analysis.

Previously the statistical analysis, a Kolmogorov-Smirnov test was conducted to assess the distribution of the variables in order to use a parametric or non-parametric tests.

With the aim to describe the study sample characteristics and to evaluate differences among patients with and without vertebral fractures a descriptive and comparative analysis was done on patients' sociodemographic characteristics (age, education level) and clinical variables (Body Mass Index (BMI), number of years with menopause, presence or absence of non-vertebral fractures, concomitant diseases and received treatment during the previous year) according to vertebral fracture presence. In order to compare the two groups, the chi-squared test was used, given that the variables were categorical, and the Bonferroni correction for multiple tests.

The feasibility of the ECOS-16 was analysed on the basis of missing data, time and method of administration. In order to evaluate the missing data, the number and percentage of missing response items in the whole questionnaire as well as the number and percentage of patients who failed to respond to any of the questionnaire's items were both calculated. The time spent administering the questionnaire was evaluated according to the method of administration (self-administered or administered by an interviewer).

The floor (percentage of patients with the lowest score) and ceiling (percentage of patients with the highest score) effect were calculated for each one of the ECOS-16 items and for the overall score.

An exploratory factor analysis was used to assess the content validity of the ECOS-16, using the scores obtained during the baseline by all patients for all 16 questionnaire items. Factors were extracted using principal-axis factoring method and varimax rotations. The adequacy of the factor analysis was assessed with the Kaiser-Meyer-Olkin measure and the Bartlett's test of sphericity [15].

To evaluate the construct validity, the correlations between the ECOS-16 scores and the patients' sociodemographic and clinical characteristics (bivariate analysis) were analysed. Pearson's correlation coefficient was used for continuous variables and the analysis of variance (ANOVA) for categorical variables. A multivariate analysis was also carried out taking the ECOS-16 score as a dependent variable and the sociodemographic and clinical variables that were significant in the bivariant analysis as independent variables, so that any possible confounding factors could be controlled. Secondly, the relationship between the ECOS-16 and the EQ-5D scores, the four 7-point items (general health status, back pain, limitation in daily activities and emotional status) and the MINI-OQLQ (only outpatients) were all analysed using Spearman's correlation coefficient, apart from the VAS which was analysed using Pearson's correlation coefficient. Higher correlations were expected between dimensions that measure the same HRQoL aspects.

Because the lowest score in ECOS-16, in EQ-5D and in 7-point general health status item represent the best HRQoL, the expected correlations among their dimensions would be positive. The opposite applies to the dimensions of MINI-OQLQ and the other three 7-point items (back pain, limitation in daily activities and emotional status), where the lowest score represent the worst HRQoL and, therefore, the expected correlations between ECOS-16 would be negative.

The reliability of the ECOS-16 was evaluated in terms of internal consistency and test-retest reliability. Internal consistency was calculated by Cronbach's α coefficient using the baseline scores of all questionnaire items. Test-retest reliability was evaluated only for outpatients who did not perceive a change in their general health status due to osteoporosis after a month, as shown by the change in 7-point general health status item (response category: 'More or less the same'). The Intraclass Correlation Coefficient (ICC) between the scores for both visits was used for this analysis. The hypothesis that the standard psychometric recommendations for Cronbach's α and ICC were greater than or equal to 0,7 was taken as a starting point for both internal consistency and test-retest reliability [16].

Longitudinal validity of the ECOS-16 was evaluated by analysing the correlations among changes registered in the ECOS-16, and changes in the EQ-5D, changes in the MINI-OQLQ and changes in the four 7-point change items from baseline to visit at 6 months. For this purpose the Spearman's correlation coefficient was used. The expected correlations are the same as in the construct validity.

To assess responsiveness to change, first of all, attention has been drawn to whether the questionnaire detects the changes which are perceived by patients between the baseline and the visit at 6 months. In order to do so, the Student's t-test for paired data was used. To assess the magnitude of changes, the effect size was calculated, thus it was establish that the changes in the patients' scores increased at the same time that the changes perceived by patients. In order to calculate the effect size, the change in 7-point general health status item is used [17].

The Minimal Clinically Important Difference (MCID) has been defined as the smallest difference between the scores in a questionnaire that the patient perceives to be beneficial [14]. The MCID was calculated for those patients who, at visit at 6 months, declared changes "slightly better" in the general health status item (difference between the scores from baseline and the visit at 6 months).

## Results

Table 1 shows the patients' sociodemographic and clinical characteristics evaluated according to the presence of vertebral fractures.

**Table 1:** Patients' sociodemographic and clinical characteristics according to the presence of vertebral fracture

	**With vertebral fracture**	**Without vertebral fracture**	**Total**
*Age*^‡^			
≤ 65 years	42 (32.8%)	92 (51.4%)	134 (43.6%)
> 65 years	86 (67.2%)	87 (48.6%)	173 (56.4%)
Total	128 (100.0%)	179 (100.0%)	307 (100.0%)
*Education level*			
No formal education	35 (28.2%)	46 (25.7%)	81 (26.7%)
Primary school	78 (62.9%)	101 (56.4%)	179 (59.1%)
Secondary school	11 (8.9%)	27 (15.1%)	38 (12.5%)
University	---	5 (2.8%)	5 (1.7%)
Total	124 (100.0%)	179 (100.0%)	303 (100.0%)
*BMI*^†^			
≤ 30	94 (73,4%)	153 (83,6%)	247 (79,4%)
> 30	34 (26,6%)	30 (16,4%)	64 (20,6%)
Total	128 (100,0%)	183 (100,0%)	311 (100,0%)
*Years with menopause*^‡^			
≤ 20 years	59 (46.8%)	115 (65.3%)	174 (57.6%)
> 20 years	67 (53.2%)	61 (34.7%)	128 (42.4%)
Total	126 (100.0%)	176 (100.0%)	302 (100.0%)
*Non-vertebral fractures*			
Presence	24 (18.7%)	21 (11.4%)	45 (14.4%)
Absence	104 (81.3%)	163 (88.6%)	267 (85.6%)
Total	128 (100.0%)	184 (100.0%)	312 (100.0%)
*Concomitant diseases*^‡^			
Presence	81 (62.8%)	144 (79.1%)	225 (72.3%)
Absence	48 (37.2%)	38 (20.9%)	86 (27.7%)
Total	129 (100.0%)	182 (100.0%)	311 (100.0%)
*Received treatment In the previous year*^‡^			
Yes	92 (70.2%)	71 (38.4%)	163 (51.6%)
No	39 (29.8%)	114 (61.6%)	153 (48.4%)
Total	131 (100.0%)	185 (100.0%)	316 (100.0%)

^†^p < 0.05 ^‡ ^p < 0.007

Statistically significant differences (p < 0.01) were found for age, concomitant diseases (patient-reported), and length of time with menopause, it being the case that those patients with vertebral fractures were older and had had menopause for a longer time. In addition, those patients with vertebral fracture were those that had received more treatments during the previous year (p < 0.01) and also had a greater BMI (p < 0.05). However, those patients without a vertebral fracture presented concomitant diseases more frequently (p < 0.01). Only 14.4% of patients showed some type of non vertebral fracture and about 70% had completed at least primary education level.

After the Bonferroni correction, the analysis that were still significant (p < 0,007) were age, years with menopause, concomitant disease and previous treatment.

At baseline, all osteoporotic patients were prescribed or changed their treatment by the physicians, some having changed his previous treatment but others having received no treatment before.

The mean (SD) administration time of the questionnaire was 12.3 (7.8) minutes for all patients, with a median of 10 minutes. In 55.1% of cases, the questionnaire was self-administered, the remainder being administered by health care personnel (due to eyesight difficulties or illiteracy). No statistically significant differences were observed in administration times according to the type of administration.

The 99.7% of the patients answered all items of the questionnaire. Only one patient failed to respond to any item.

A third of the items had a floor effect greater than or equal to 20%, 38.3% in item "Do you have problems with dressing?". The maximum ceiling effect was observed in 55.7% of the patients in item "How often have you had back pain in the last week?". In the remaining items, the ceiling effect was lower, ranging from 1.3% to 20.3%, the highest being in item "Are you afraid of getting a fracture?". Two patients (0.7%) recorded the highest scores for all the items (ceiling effect of the overall score).

In the factor analysis, the Kaiser-Meyer-Olkin measure was 0.916 indicating a good sampling adequacy. The Bartlett's test of sphericity (p < 0.001) made it possible to accept the identity of the matrix correlations for the ECOS-16 items, thus indicating the suitability of the factor analysis.

Table 2 shows the relationship between the patients' sociodemographic and clinical characteristics and the ECOS-16 score. In the bivariate analysis was observed a worse HRQoL in women with greater BMI (p < 0.05), a lower education level (p < 0.01) and concomitant chronic diseases (p < 0.05).

**Table 2:** ECOS-16 scores according to patients' clinical and sociodemographic characteristics

	**N**	**ECOS-16 mean (SD) score**
*Age*		
≤ 65 years	134	2.79 (0.77)
> 65 years	173	2.92 (0.82)
Total	307	2.87 (0.80)
*Education level*^‡^		
No formal education	81	3.15 (0.73)
Primary school	179	2.84 (0.81)
Secondary school	38	2.46 (0.63)
University	5	1.94 (0.58)
Total	303	2.86 (0.80)
*BMI*^†^		
≤ 30	247	2.81 (0.79)
> 30	64	3.06 (0.81)
Total	311	2.86 (0.80)
*Years with menopause*		
≤ 20 years	174	2.80 (0.78)
> 20 years	128	2.95 (0.83)
Total	302	2.86 (0.81)
*Vertebral fractures*		
Presence	131	2.94 (0.83)
Absence	185	2.81 (0.78)
Total	316	2.86 (0.80)
*Non-vertebral fractures*		
Presence	45	2.88 (0.71)
Absence	267	2.87 (0.81)
Total	312	2.87 (0.80)
Lumbar BMD*	298	0.024
Lumbar T-score *	308	-0.004
Neck BMD*	257	-0.042
Neck T-score*	264	0.090
*Concomitant diseases*^†^		
Presence	225	2.91 (0.81)
Absence	86	2.71 (0.72)
Total	311	2.85 (0.79)
*Received treatment in the previous year*		
Yes	163	2.87 (0.77)
No	153	2.86 (0.83)
Total	316	2.86 (0.80)

*Pearson's correlation coefficient ^† ^p < 0.05 ^‡ ^p < 0.01

A multivariate analysis was carried out to identify patients' characteristics that were related to the ECOS-16 score (the variables entered into the multivariate analysis were age, education level, BMI, years with menopause, non-vertebral fractures, concomitant diseases and received treatment in previous years, all were codified as in table 1). The results showed that the variables of education level, number of concomitant diseases and the presence of vertebral fractures were related to the ECOS-16 score. Nevertheless, the percentage of variance explained by the variables included in the multivariate model was low, 11.1%.

Table 3 shows the relationship between the ECOS-16 score and each one of the EQ-5D's dimensions, the four 7-point items and the MINI-OQLQ. All the EQ-5D's dimensions showed a statistically significant correlation with the ECOS-16 score, the dimensions with the greatest correlation being 'Mobility', 'Self-Care' and 'Pain/Discomfort'. The VAS was also statistically significant, with a Pearson's correlation coefficient of 0.61. The four 7-point items showed high correlations (greater than 0.7) with the ECOS-16 score, the item 'Limitation in daily activities' being the highest correlated item (0.82). The MINI-OQLQ dimensions showed moderate but statistically significant correlations (range: 0.47–0.73) with the ECOS-16, the 'Symptoms' and 'Leisure' dimensions having the highest correlations (0.71 and 0.74, respectively).

**Table 3:** Correlations between the ECOS-16 scores and the EUROQoL-5D, the four 7-point items scores and the MINI-OQLQ

	**N**	**Spearman's correlation**
*EUROQol-5D*		
Mobility	315	0.643
Self-care	315	0.623
Usual activities	315	0.594
Pain/Discomfort	314	0.608
Anxiety/Depression	315	0.555
VAS^a^	312	-0.610
*General health status*	316	0.712
*Back pain*	313	-0.741
*Limitation in daily activities*	313	-0.822
*Emotional status*	313	-0.791
*MINI-OQLQ *^b^		
Symptoms	104	-0.711
Physical function	104	-0.670
Activities of daily living	104	-0.455
Emotional function	104	-0.473
Leisure	104	-0.736

^a^Pearson's Correlation Coefficient; ^b^Outpatients only; All correlations are statistically significant at the 0.001 level

The observed correlations between the changes among ECOS-16 questionnaire and the changes among the dimensions of the HRQoL questionnaires administered: EQ-5D, MINI-OQLQ and the four 7-point change items were similar to those of the construct validity (range: 0.33–0.76). All these correlations were also statistically significant.

The internal consistency of the ECOS-16 was very high, with a Cronbach's α coefficient of 0.92. Test-retest reliability was analysed for 44 outpatients who declared that their general health status due to osteoporosis had not changed after a month, with an Intraclass Correlation Coefficient of 0.80 and a mean (SD) score change of 0.2 (0.5) points.

**Figure 1:**
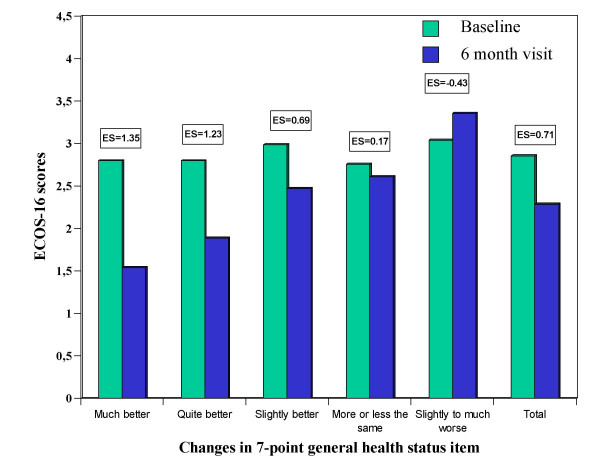
ECOS-16 scores according to perceived changes in general health status

Figure 1 shows the ECOS-16 patient scores for baseline and at visit at 6 months, as well as the effect size (ES) according to the changes in 7-point general health status item perceived by the patient after 6 months. The greater the perception of change in patients' general health status, the greater the changes in patients' scores. The same happened with the effect size as the patients who declared a 'much better' change in their general health status due to their osteoporosis at visit at 6 months had an effect size of 1.35, compared to those patients who declared a 'quite better' change having an ES of 1.23. The Minimal Clinically Important Difference (MCID) represented a mean change (SD) in the ECOS-16 score of 0.69 points, taking the category representing the least improvement in general health status due to their osteoporosis: 'slightly better'.

## Discussion

The measurement of HRQoL has attracted increasing attention as a clinically relevant outcome of research and clinical practice. HRQoL questionnaires reflect the impact of health care interventions on health aspects such as physical, mental and social well-being. However, either in clinical research and in practice, a lengthy questionnaire is problematic for both the health care personnel and the patient. Shortish measure attempt to minimize time and effort as well as to increase patient interest [18]. Thus, shortish questionnaires need to be sufficiently psychometrically robust, proving that they are truly measuring what they set out to (validity), that they measure in a reliable way (reliability) and that they are capable of detecting real changes in perceived health status among patients with osteoporosis (responsiveness to change).

The ECOS-16 originates from the reduction of two validated and widely used HRQoL questionnaires in osteoporosis patients with vertebral fracture [11]. However, in the present study, the ECOS-16 was administered to as many patients with fracture as those without, in order to establish its general applicability to patients with osteoporosis. Although the ECOS-16 requires a short time to be administered, self-administration was not possible in a high percentage of patients who needed help from health care personnel under conditions of usual clinical practice. In the future, it would be necessary to assess whether scores obtained through the questionnaire are maintained once the administration method changes, which would give more consistency to the questionnaire.

Almost all the patients responded to all the questionnaire items. The presence of a floor effect for one third of the questionnaire items could lead to the conclusion that the questionnaire detects changes only when the severe disease status occurs. However, according to a previous qualitative division [12], the floor effect is concentrated both in the items belong to physical and psychosocial dimensions as for the mobility and self-care EQ-5D dimensions -the most conceptually equivalent- leading to think that the study sample has a certain clinical stability.

As some previous studies, this study shows that the variables usually used to evaluate patients with osteoporosis, such as Bone Mineral Density (BMD) and the presence of vertebral fractures, have low or no correlation with HRQoL scores. This finding is not new [19,20] and suggests that HRQoL scores could be influenced by other factors such as personal, clinical and sociodemographic characteristics. In this study, the fact that patient's education level is the most significantly correlated variable draws particular attention. However, this observation has already been made in previous studies in patients with musculoskeletal problems [21,22]. The presence of concomitant diseases and/or a higher Body Mass Index (BMI) is scarcely correlated with HRQoL. This finding is interesting when taking into account studies evaluating HRQoL in patients with osteoporosis, whether they be descriptive or experimental in its design. The outcomes between compared groups could be underestimated if potential characteristic differences, particularly the education level, are not monitored. In this respect, the ECOS-16 apparently remains scientifically robust in its ability to discriminate among different education levels.

In this study, the fact that the presence of vertebral fractures does not have a negative effect on HRQoL (bivariate analysis) deserves special attention. There seems to be some discrepancy regarding this issue in literature. Several studies have shown that HRQoL progressively deteriorates in relation to the presence and number of vertebral fractures [23,24]. However, other studies in the same area failed to find such a relationship [25], or have only found it when vertebral deformity is severe, while failing to find a relationship with any other fractures [26,27].

Recently, a significant number of studies have highlighted the importance of the site of the vertebral fracture and its effect on HRQoL. In this regard, it seems that the site of vertebral fracture has a much greater effect on HRQoL than the presence and number of vertebral fractures [28-30]. This difference could be explained by the relative rigidity of the thorax column in relation to the lumbar column, in the sense that mobility is more restricted when a lumbar rather than a thoracic region fracture occurs [31]. Moreover, lumbar column deformities have probably a greater impact on postural stability than alterations to the thoracic column. When the severity and site of the fracture is taken into account, fractured vertebrae in the transitional thoracolumbar region have a negative impact on HRQoL, at a Genant's degree greater than 1 [32]. Nevertheless, prospective studies addressing this issue should be conducted in the future assessing the impact of the time of the fracture and the site.

Although in the present study, the bivariate analysis does not discriminate between patients with and without vertebral fracture, the multivariate model shows that the presence of fractures is indeed significant even if the percentage of explained variation is small. This is not surprising given the fact that such analysis is dealing with prevalent fractures in a short term observational study [28]. The inclusion of the time spent since the fracture occurred could improve the model, since it is well known that pain and disability due to a fracture progressively diminish over time [33]. However, among other health problems, a small percentage of explained variance was also found [33]. Nevertheless, the variables were entered into the multiple regression analysis dichotomously, as in table 1, an this may reduce the likelihood of finding a relationship. Therefore, it is likely that administering a HRQoL questionnaire in conjunction with the analysis of clinical variables could provide a better overall picture of the osteoporosis impact on patients.

The results obtained for internal consistency and test-retest reliability showed high levels of homogeneity among questionnaire items and good reproducibility over time. Moreover, the outcomes, have also been shown that the new questionnaire effectively detects changes in patients' perceived health status due to osteoporosis. Expressing responsiveness to change is an important characteristic of the new instrument, one which will doubtless allow its use in clinical research. The high correlation between the ECOS-16 and generic (EQ-5D) and specific (MINI-OQLQ) HRQoL questionnaires corroborates this hypothesis. It also highlights the new questionnaire's validity by demonstrating that it measures concepts which are closely related to already validated HRQoL questionnaires [3,13].

The mean change in score per question corresponding to the effect size in general and to the MCID in particular is consistent, in terms that the larger the change assessed by the ECOS-16, the larger the effect size. Moreover, MCID is consistent with the results of other HRQoL questionnaires, and it is useful to compare the magnitude of changes detected between them. MCID will also be useful in the planning of new trials, as sample size depends on the magnitude of the difference investigators consider clinically important and are not willing to risk failing to detect [34].

The potential limitations of this study are mainly due to it being unable to rely on certain variables which have shown a clear influence on HRQoL. In particular, the "time spent since the fracture occurred" [35] was not analysed even though the objective of the study were women with established osteoporosis. The inclusion of prevalent fractures and exclusion of the incidence fractures means that a smaller variability among the patients in this study was established and, possibly, it also means that vertebral fractures had less influence on HRQoL. A further possible limitation is the limited sample size, which was relatively low to obtain statistical significance for more than one fracture site and for a certain fracture severity. This was a prospective, observational study with a limited follow-up time, but under conditions of usual clinical practice it served to prove the short-term good responsiveness of the questionnaire and test its remaining psychometric properties. Nevertheless long term follow-up studies will be necessary in the future.

The low education level of the study sample must also be taken into account. It is consistent with previous studies [8,9], and as in other chronic diseases, education level has an impact on the prevalence of osteoporosis [36] and on the preferences of patients [37]. Furthermore, the impact of osteoporosis on HRQoL, the site of the vertebral fracture, coupled with the time spent since its onset, are extremely important variables which must be taken into account, since traditional clinical variables -i.e. bone densitometry- do not have a remarkable relationship with HRQoL [38].

## Conclusions

In conclusion, the ECOS-16 is a HRQoL questionnaire which is short, easy to administer (although some women need aid) and with adequate preliminary psychometric properties. This makes the ECOS-16 potentially very useful during routine clinical practice or/and research for the treatment and follow-up of post-menopausal women with osteoporosis. Nevertheless, its actual potential must be proven in future clinical trials in order to recommend its use in research and clinical practice.

## Supplementary Material

Additional File 1 ECOS Appendix1.doc, the ECOS-16 questionnaire Click here for file
