# Supplementary material for: The ECOS-16 questionnaire for the evaluation of health related quality of life in post-menopausal women with osteoporosis
Source: Health Qual Life Outcomes. 2004 Aug 3;2:41. doi: 10.1186/1477-7525-2-41 (PMC514569; doi:10.1186/1477-7525-2-41)
Supplement: Additional File 1 — ECOS Appendix1.doc, the ECOS-16 questionnaire [file 1477-7525-2-41-S1.doc]

Appendix

| ECOS-16 QUESTIONNAIRE |
| --- |

**During the last week and because of your back problems due to osteoporosis,**

1. How often have you had back pain in the last week?

|  | 1. I have not had back pain |
| --- | --- |
|  |  |
|  | 1. 1 day |
|  |  |
|  | 1. 2-3 days |
|  |  |
|  | 1. 4-6 days |
|  |  |
|  | 1. Every day |

1. How severe is your back pain?

|  | 1. I have not had back pain |
| --- | --- |
|  |  |
|  | 1. Mild |
|  |  |
|  | 1. Moderate |
|  |  |
|  | 1. Severe |
|  |  |
|  | 1. Intolerable |

1. How much distress or discomfort have you had because it has been painful to stand for a long time?

|  | 1. No discomfort or suffering |
| --- | --- |
|  |  |
|  | 1. Slight discomfort or suffering |
|  |  |
|  | 1. Moderate discomfort or suffering |
|  |  |
|  | 1. Severe discomfort or suffering |
|  |  |
|  | 1. Very severe discomfort or suffering |

1. How much distress or discomfort have you had due to pain from bending?

|  | 1. No discomfort or suffering |
| --- | --- |
|  |  |
|  | 1. Slight discomfort or suffering |
|  |  |
|  | 1. Moderate discomfort or suffering |
|  |  |
|  | 1. Severe discomfort or suffering |
|  |  |
|  | 1. Very severe discomfort or suffering |

1. Has the back pain disturbed your sleep in the last week?

|  | 1. On no occasion |
| --- | --- |
|  |  |
|  | 1. One night |
|  |  |
|  | 1. Two nights |
|  |  |
|  | 1. Three or four nights |
|  |  |
|  | 1. Every night |

1. How difficult has it been for you to carry out the household activities?

|  | 1. No difficulty |
| --- | --- |
|  |  |
|  | 1. Slight difficulty |
|  |  |
|  | 1. Moderate difficulty |
|  |  |
|  | 1. Great difficulty |
|  |  |
|  | 1. I was unable to do anything |

1. Can you climb stairs to the next floor of a house?

|  | 1. No difficulty |
| --- | --- |
|  |  |
|  | 1. Slight difficulty |
|  |  |
|  | 1. I had to rest at least once |
|  |  |
|  | 1. I could only climb the stairs with help |
|  |  |
|  | 1. I was unable to climb the stairs |

1. Do you have problems with dressing?

|  | 1. No difficulty |
| --- | --- |
|  |  |
|  | 1. I can dress myself with slight difficulty |
|  |  |
|  | 1. I can dress myself with moderate difficulty |
|  |  |
|  | 1. I sometimes need help to dress myself |
|  |  |
|  | 1. I cannot dress myself unaided |

1. How difficult has it been for you to bend?

|  | 1. No difficulty |
| --- | --- |
|  |  |
|  | 1. Slight difficulty |
|  |  |
|  | 1. Moderate difficulty |
|  |  |
|  | 1. Great difficulty |
|  |  |
|  | 1. I am unable to bend down |

1. How much has your walking been limited?

|  | 1. Not limited |
| --- | --- |
|  |  |
|  | 1. Slightly limited |
|  |  |
|  | 1. Moderately limited |
|  |  |
|  | 1. Very limited |
|  |  |
|  | 1. I am unable to walk |

1. How difficult has it been for you to visit friends or relatives?

|  | 1. No difficulty |
| --- | --- |
|  |  |
|  | 1. Slight difficulty |
|  |  |
|  | 1. Moderate difficulty |
|  |  |
|  | 1. Great difficulty |
|  |  |
|  | 1. I have been unable to visit family or friends |

1. Do you feel downhearted?

|  | 1. No |
| --- | --- |
|  |  |
|  | 1. Rarely |
|  |  |
|  | 1. Sometimes |
|  |  |
|  | 1. Often |
|  |  |
|  | 1. Always |

1. Are you hopeful about your future?

|  | 1. Always |
| --- | --- |
|  |  |
|  | 1. Often |
|  |  |
|  | 1. Sometimes |
|  |  |
|  | 1. Rarely |
|  |  |
|  | 1. No |

1. Do you feel frustrated?

|  | 1. No |
| --- | --- |
|  |  |
|  | 1. Rarely |
|  |  |
|  | 1. Sometimes |
|  |  |
|  | 1. Often |
|  |  |
|  | 1. Always |

1. Are you afraid of falling?

|  | 1. No |
| --- | --- |
|  |  |
|  | 1. Rarely |
|  |  |
|  | 1. Sometimes |
|  |  |
|  | 1. Often |
|  |  |
|  | 1. Always |

1. Are you afraid of getting a fracture?

|  | 1. No |
| --- | --- |
|  |  |
|  | 1. Rarely |
|  |  |
|  | 1. Sometimes |
|  |  |
|  | 1. Often |
|  |  |
|  | 1. Always |
